# Enterosalpingeal fistula complicating Crohn's disease: Report of two cases and review of the literature

**DOI:** 10.1016/j.amsu.2021.102734

**Published:** 2021-08-17

**Authors:** A. Haddad, A. Sebai, H. Maghrebi, Y. Chaker, M. Jouini, M. Kacem

**Affiliations:** Surgery Department A – La Rabta Hospital of Tunis, University of Tunis El Manar, Faculty of Medicine of Tunis, Tunisia

**Keywords:** Crohn's disease, Enterosalpingeal fistulae, Case report

## Abstract

**Introduction and importance:**

Reports of enterosalpingeal fistulae complicating Crohn's disease are scarce. They involve the last ileal loop and lead to a progressive destruction of the salpinx. Usually, no genital symptoms are found. In all the cases reported in the literature, the fistula was diagnosed intra-operatively and resection of the right salpinx was performed without the patient's pre-operative consent.

**Case presentation:**

We describe 2 cases of women presenting with an Enterosalpingeal fistulae complicating Crohn's disease. Radiological findings allowed a pre-operative diagnosis. Thus, the patients were warned of the right salpinx resection and consent was obtained.

**Clinical discussion:**

Enterosalpingeal fistulae complicating Crohn's disease are exceptional. Indeed, to the best of our knowledge, only five cases have been reported till now. In all the reported cases, no genital signs were present. As for our patients who didn't experience such symptoms. Moreover, no radiological evidence of the enterosalpingeal fistula was found in the literature. Consequently, the fistula was always diagnosed intra-operatively. For our patients, radiological findings allowed a pre-operative diagnosis. This permitted to warn them of a possible resection of the fallopian tube. Intra-operative findings were unfortunately conflicting with its preservation.

**Conclusion:**

Enterosalpingeal fistula is an exceptional complication of the Crohn's disease. No clinical findings are present. The diagnosis should be evoked when the CT-scan or the MRI show an abnormal apposition between the fallopian tube, the last loop and the cecum. Surgical resection of the involved salpinx with the diseased intestinal segment is unfortunately usually needed in a young patient population.

## Highlights

• Report of two cases of an unusual form of Crohn's disease.
• Unique preoperative imaging.
• Preoperative diagnosis of the fistulae. Thus, patients were warned of salpingectomy.

## 1 Introduction

Fistulae represent a common complication of Crohn's disease that occurs in about 30% of patients [1]. The most frequent fistulae are enterocolic and enteroenteric [1]. Reports of enterogenital fistulae are scars. Herein, we present two cases of enterosalpingeal fistulae complicating Crohn's disease in young women. Radiological findings allowed a pre-operative diagnosis. Thus, the patients were warned of the right salpinx resection and consent was obtained.

This case report has been reported in line with the SCARE Criteria [2].

## 2 Case 1

**Fig. 1:**
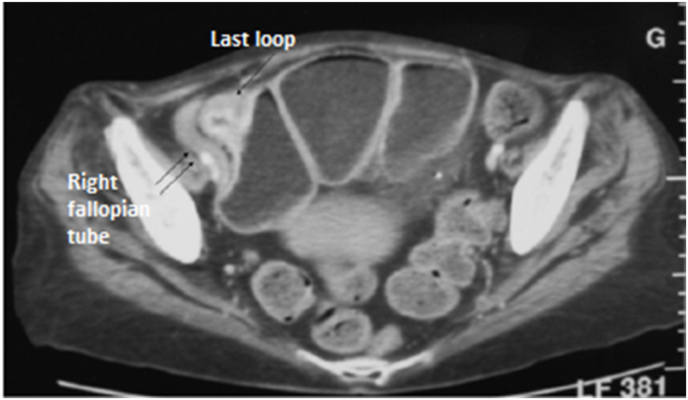
Thickened right fallopian tube having an abnormal contact with the last loop.

**Fig. 2:**
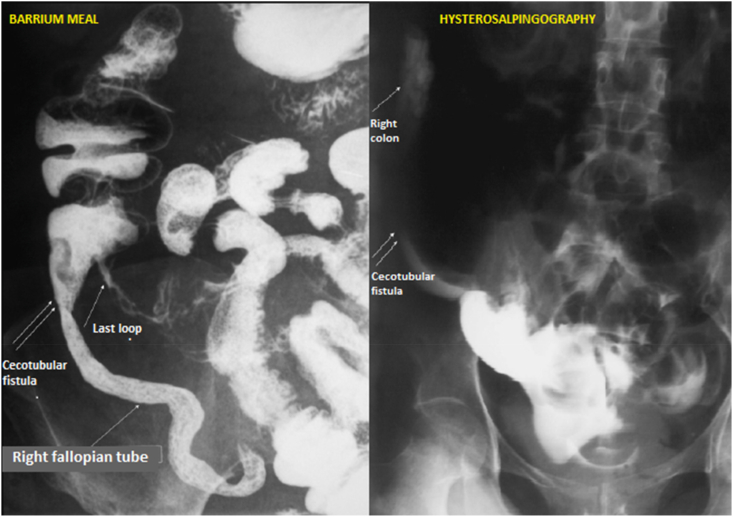
Barium meal and hysterosalpingography exposing the fistula as contrast fluid reached the right fallopian tube and the cecum in both examinations.

She was a fifty-year-old woman, with a past medical history of ileocecal Crohn's disease evolving since three years. She was admitted in our department for bowel obstruction. On physical examination, the patient had a mass in the right fossa iliaca. Routine serological tests were normal. Abdominal CT-scan **(**Fig. 1**)** showed a severe stenosis of the last ileal loop causing intestinal distension. Sclerolipomatosis and mesenteric adenopathies were present. CT also showed a markedly thickened right fallopian tube having an abnormal contact with the last loop. Conservative management was successfully conducted. Barium meal **(**Fig. 2**)** revealed a stenosis of the last loot and presence of contrast fluid in the right fallopian tube. Hysterosalpingography **(**Fig. 2**)** showed an opacification of the ascending colon. Thus, the diagnosis of last loop stenosis and enterosalpingeal fistula complicating Crohn's disease was retained. Surgery was then decided.

A median laparotomy was performed. There was a tumor-like mass of the right fossa iliaca joining the last ileal loop, the cecum and the right fallopian tube with an intense sclerolipomatosis. As dissection was highly haemorrhagic, en-bloc resection of the mass and ileocolic anastomosis were executed. Outcomes were straightforward.

## 3 Case 2

**Fig. 3:**
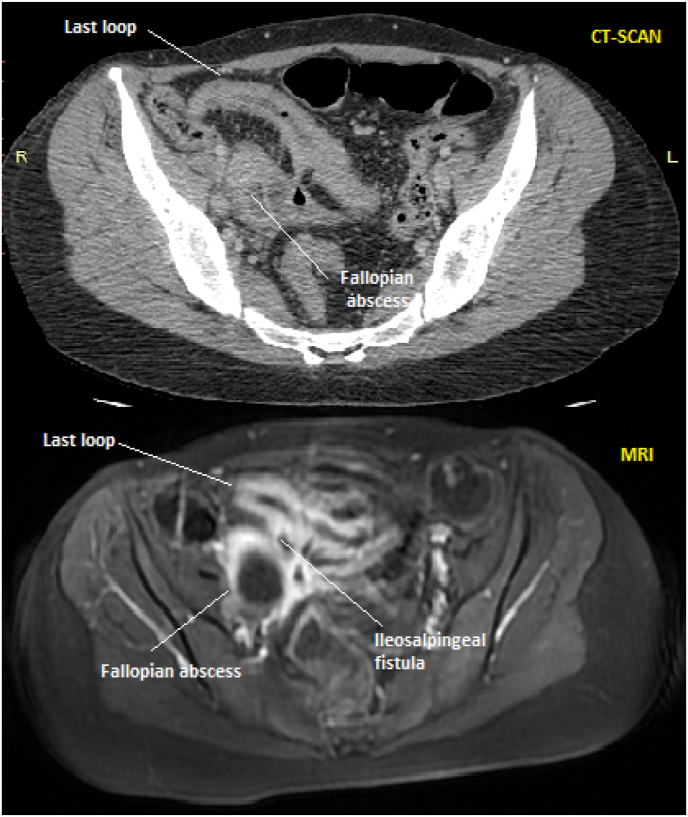
*Abdominal CT-scan* showing an abscess of the right fallopian tube that was adjoining the last loop. *Abdominal MRI* revealing an abnormal connection between the right salpinx and the last loop with T2 hypersignal.

She was a twenty-year-old patient, with no past medical history. She was admitted in our department for an appendicular syndrome. Interrogatory discovered a six-month-evolving diarrhea and a recent weight loss. On physical examination, the patient had fever, a sensitive mass and guarding of the right fossa iliaca. Serological tests found heightened white blood count (16,520/mm^3)^ and C Reactive Protein (142 mg/L). Abdominal CT-scan **(**Fig. 3**)** showed a 7 cm abscess of the right fallopian tube that was adjoining the last loop. The latter was thickened and enhanced after intravenous contrast fluid injection. Multiple coeliomesenteric adenopathies were present. Abdominal MRI **(**Fig. 3**)** found an abnormal connection between the fallopian abscess and the last loop that was highlighted in T2 sequences evoking an ileosalpingeal fistula. Colonoscopy with ileocecal biopsies confirmed the diagnosis of Crohn's disease. Therefore, the diagnosis of ileo-salpingeal fistula with right fallopian tube abscess complicating Crohn's disease was retained.

**Fig. 4:**
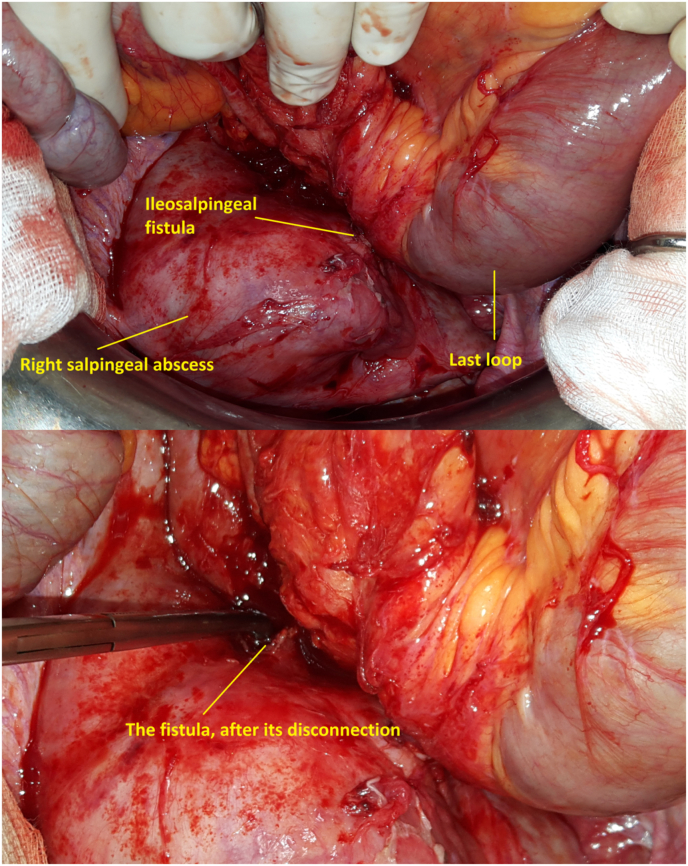
Right salpingeal abscess and ileosalpingeal fistula before and after its disconnection.

The patient was put on antibiotics. Nevertheless, she developed a severe sepsis syndrome. Emergency laparotomy was then performed. We found an ileosalpingeal fistula powering a right fallopian abscess **(**Fig. 4**)**. There also were an intense sclerolipomatosis of the right fossa iliaca and a thickened last ileal loop. After disconnection of the fistula, an ileocecal resection was performed. However, preserving the right salpinx wasn't possible, as it was too distended with a necrotic mucosae and no contractile activity. Outcomes were straightforward.

## 4 Discussion

Fistula is a common complication of the Crohn's disease. Yet, enterogenital fistulae are rare and mainly rectovaginal [3]. Enterosalpingeal fistulae complicating Crohn's disease are exceptional. Indeed, to the best of our knowledge, only five cases have been reported till now [1,4,5]. All these patients' characteristics are summarized in Table 1.

**Table 1:** Patients’ characteristics.

	Genital symptoms	Preoperative diagnosis	Involved salpinx	Involved intestine	Salpingeal abscess	Salpingeal resection
*Michelassi F. and al* [1]	No	No	Right	Last loop	No	Yes
*Michelassi F. and al* [1]	No	No	Right	Last loop	No	Yes
*Champault G. and al* [3]	No	No	Right	Last loop	No	Yes
*Champault G. and al* [3]	No	No	Right	Last loop	No	Yes
*Maun D. and al* [4]	No	No	Right	Last loop	No	Yes
*Case 1*	No	Yes	Right	Cecum	No	Yes
*Case 2*	No	Yes	Right	Last loop	Yes	Yes

Enterosalpingeal apposition with progressive perforation of the gut in the fallopian tube leads to the fistula. Likewise, intra-abdominal abscess may drain into the fallopian tube and form the fistula.

In all the reported cases, no genital signs - as leucorrhoea, dyspareunia, genital infections - were present. As for our patients who didn't experience such symptoms.

Moreover, no radiological evidence of the enterosalpingeal fistula was found in the literature. Consequently, the fistula was always diagnosed intra-operatively. Nevertheless, radiological findings allowed a pre-operative diagnosis for our two patients.

Actually, CT-scan revealed an abnormal contact between the right fallopian loop and the intestine in both cases. Besides, it diagnosed a salpingeal abscess for our second patient. Furthermore, barium meal and hysterosalpingography exposed the fistula as contrast fluid reached the right fallopian tube and the cecum in both examinations for our first patient. In addition, MRI discovered the fistula showing an abnormal connection between the right salpinx and the last loop with T2 hypersignal for our second patient.

It is to know that enterosalpingeal fistula may lead to salpingeal abscess and progressive destruction of the fallopian tube, like for our second patient. Thus, surgical treatment of the Crohn's disease should be indicated. Resection of the diseased intestinal segment with en-bloc dissection of the involved salpinx was the performed procedure in all the reported cases.

As dissection was hard and haemorrhagic due to intense sclerolipomatosis, we performed an en-bloc resection of the fallopian tube with the diseased intestine for our first patient, especially that she was fifty-year-old. But, salpingeal preservation was initially meant for our second patient, knowing that she was only twenty-year-old with no children. Pre-operative diagnosis permitted though to warn her of a possible resection of the fallopian tube. Intra-operative findings were unfortunately conflicting with its preservation.

## 5 Conclusion

Enterosalpingeal fistula is an exceptional complication of the Crohn's disease. No clinical findings are present. The diagnosis should be evoked when the CT-scan or the MRI show an abnormal apposition between the fallopian tube, the last loop and the cecum. Barium meal, hysterosalpingography and MRI may visualize the fistula. Surgical resection of the involved salpinx with the diseased intestinal segment is unfortunately usually needed in a young patient population.

## Declaration of interest

The authors declare that they have no conflict of interest.

## Funding source

No source has funded this manuscript.

## Ethical approval

All the authors have read and complied with the policy of the journal on ethical consent.

## Consent

Written informed consent was obtained from the patient for publication of this case report and accompanying images. A copy of the written consent is available for review by the Editor-in-Chief of this journal on request.

## Provenance and peer review

Not commissioned, externally peer-reviewed.

## Author contribution

Haddad Anis: Writing – original draft. Sebai Amine: Writing – original draft. Maghrebi Houcine: Study concept and design. Chaker Youssef: Methodology. Mohamed Jouini: Supervision. Montasser Kacem: Supervision.

## Guarantor

Sebai Amine.

## Registration of research studies

Not appliable
